# Preoperative Embolization of a Delayed Femoral Metastasis of Renal Cell Carcinoma: A Case Report

**DOI:** 10.7759/cureus.23788

**Published:** 2022-04-03

**Authors:** Christopher M Stevens, Deven Champaneri, Daniel Harper, Assala Aslan, Kevin Malone, Aliaksandr Aksionau, Naveen K Gunji

**Affiliations:** 1 Interventional Radiology, Louisiana State University Health Sciences Center, Shreveport, USA; 2 Radiology, Edward Via College of Osteopathic Medicine (VCOM) - Carolinas, Spartanburg, USA; 3 Radiology, Louisiana State University Health Sciences Center, Shreveport, USA; 4 Biomedical Engineering, Louisiana State University Health Sciences Center, Shreveport, USA; 5 Pathology, Louisiana State University Health Sciences Center, Shreveport, USA

**Keywords:** blood loss, pre-operative embolization, bone metastasis, interventional radiology, renal cell carcinoma

## Abstract

Preoperative embolization of hypervascular bone metastasis is an effective measure for reducing blood loss during open orthopedic surgery. When the clinician is experienced with the procedure, the risks of the procedure are minimal and final outcomes are typically good.

In this study, we report a case of a 50-year-old female patient who presented with a delayed metastatic renal cell tumor in the left proximal femur one year after radical nephrectomy. The patient underwent an effective preoperative embolization, which resulted in a remarkable absence of bleeding and a successful response subsequent to surgical fixation.

## Introduction

Intraoperative blood loss is often a concern with surgical procedures of long bone metastases. A large amount of blood loss can lead to high blood transfusion requirements during the operation, severe intraoperative complications, increased operative time, and delayed wound healing, all of which can be detrimental to the patient [1]. To reduce the amount of intraoperative blood loss, preoperative embolization may be performed [2,3].

An embolization is a minimally invasive procedure performed to occlude a vessel while preserving blood flow to the surrounding areas. The procedure is performed by using image guidance to place a catheter into the target vessel that is to be blocked. Once the catheter is in the correct position, proembolic agents are placed into the catheter to occlude the vessel [4].

Preoperative embolization of vascular bone metastasis was first introduced by Feldman et al. in 1975 [5]. Since then, it has been considered an effective way to reduce intraoperative blood loss following surgical management of those tumors, particularly when arising from renal carcinoma. A recent systematic review by Geraets et al. showed that half of the studies included reported a significant reduction in blood loss and the need for transfusion in renal cell carcinoma (RCC) metastasis [6]. The complications rates related to the procedure ranged from 0% to 9%, indicating a relative safety profile [6].

This report demonstrates an interesting case in which the Interventional Radiology team was needed to perform a preoperative embolization of a metastatic tumor, prior to surgical repair of a pathological fracture. This case highlights, and adds to the existing literature, the great value that preoperative embolization can provide to a case to reduce morbidity and mortality.

## Case presentation

**Figure 1:**
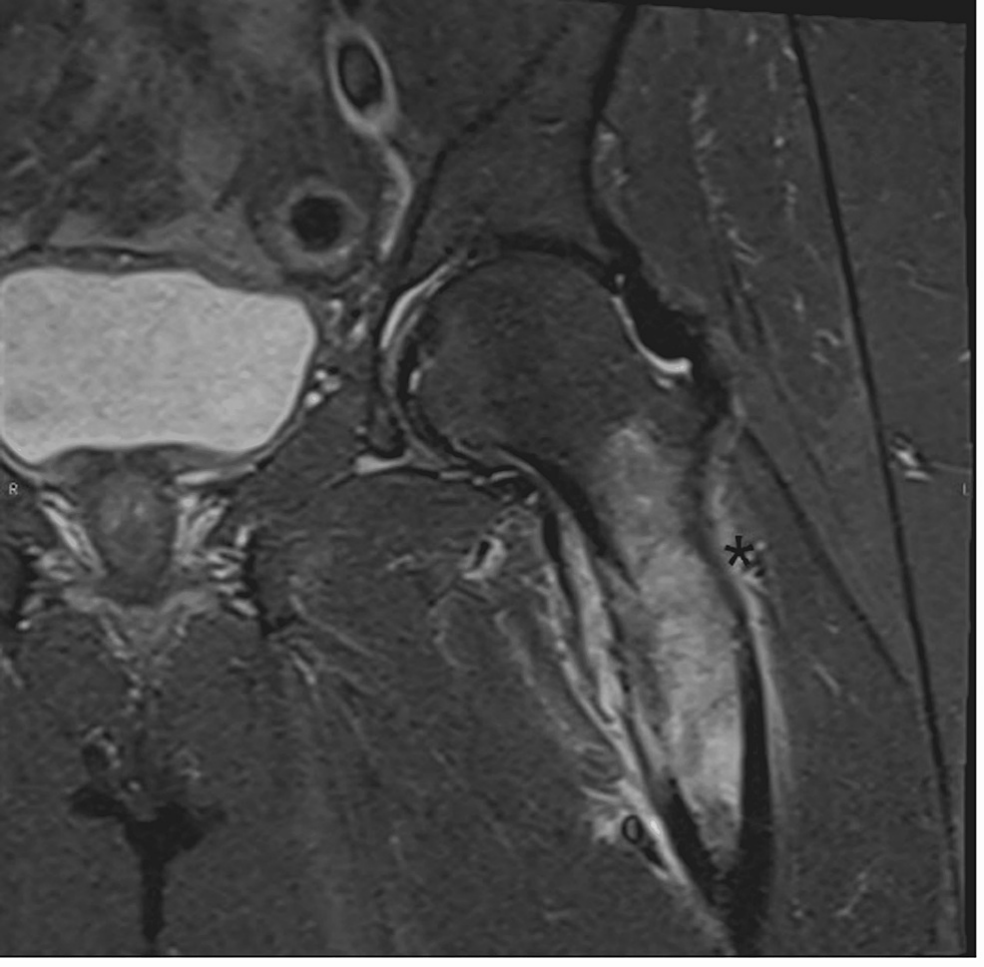
Coronal MRI of the pelvis, STIR sequence, demonstrating a destructive and infiltrating lesion in the left proximal femur (*) with periosteal reaction and bone edema. STIR, short tau inversion recovery

A 50-year-old African-American female presented to the Emergency Department for a syncopal event. The patient stated that she had a leg cramp and subsequently fell while getting out of bed. The initial workup did not rule out cardiologic or metabolic etiologies. Patient history was positive for a 10-year smoking history and family history of breast cancer. CT angiogram of the chest, abdomen, and pelvis revealed a 9.1 x 7.8 cm mass arising from the inferior pole of the right kidney with no signs of metastasis. The decision was made to perform a right radical nephrectomy. One year later, the patient presented to a family doctor following a fall. She reported new left hip pain, which radiated to the groin and knee, causing difficulty in walking or sleeping. Imaging was not obtained during that encounter. The patient returned to the family doctor two months later with a complaint of persistent and worsening left hip pain. Initial radiographs were negative for any acute findings. Outpatient MRI was ordered, which was consistent with suspected metastasis to the left proximal femur with cortical involvement and a high risk for fracture (Figure 1). CT of the chest, abdomen, and pelvis showed multiple pulmonary and skeletal lesions, including the T1 vertebral body and left femur. Subsequent pulmonary biopsies were negative for renal cell metastasis.

**Figure 2:**
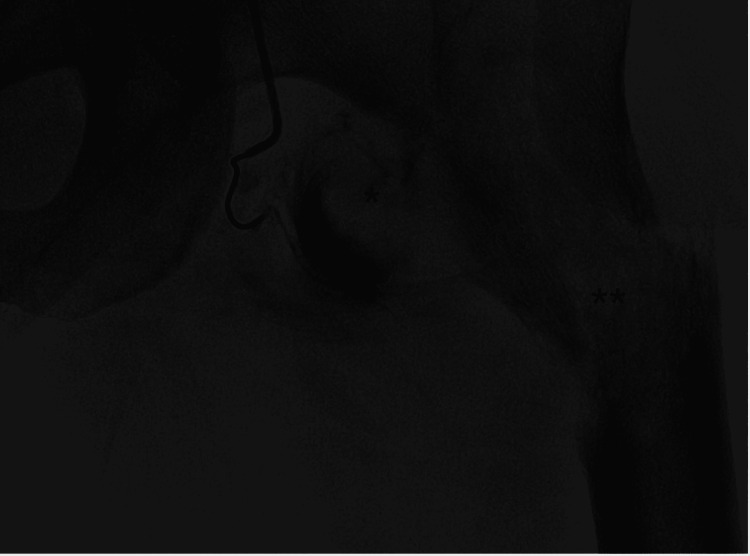
Fluoroscopic intraoperative image of the left femur showing no residual opacification of feeding vessels (*) to the metastatic lesion. The pathologic left proximal femur fracture (**) is also well seen.

**Figure 3:**
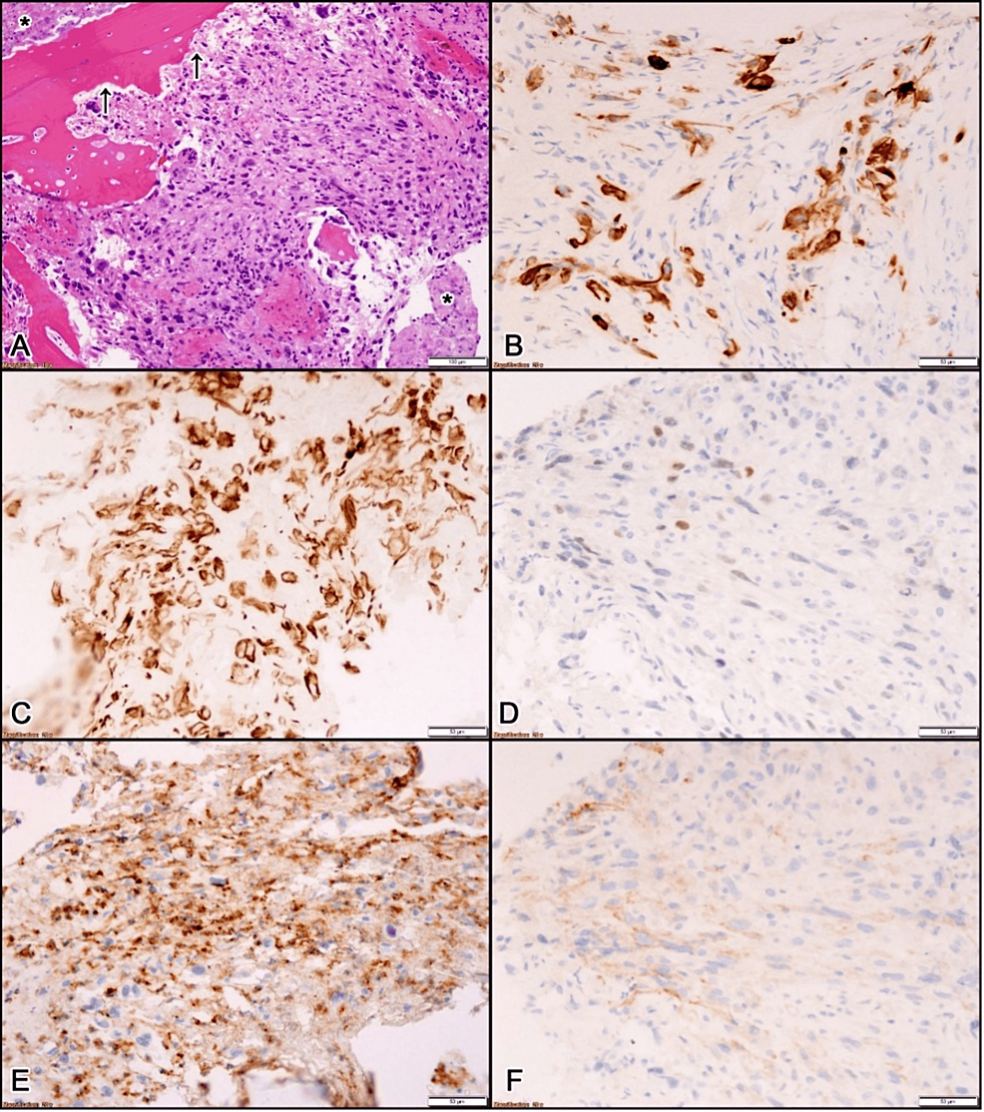
Histopathological finding of the biopsied lesion. (A) Hematoxylin and eosin staining of the tumor cells. A microphotograph represents focally necrotic (*) sheets of pleomorphic epithelioid tumor cells invading the bone (→) and replacing the bone marrow, which is not present. The nuclei of tumor cells are hyperchromatic, irregular-shaped with focally prominent nucleoli. Multinucleated osteoclasts (►) are seen near the rim of the bone. (Hematoxylin and Eosin staining, 100x). (B) AE1/AE3 immunostaining of the tumor cells. The tumor cells show focally strong cytoplasmic positivity for pancytokeratin supporting epithelial differentiation (AE1/AE3 immunostaining, 200x). (C) CAM 5.2 immunostaining of the tumor cells. The tumor cells show diffusely strong cytoplasmic positivity for low molecular weight cytokeratin, further confirming the epithelial nature of the tumor cells (CAM 5.2 immunostaining, 200x). (D) PAX-8 immunostaining of the tumor cells. The urothelial origin of the tumor cells is confirmed by focally strong nuclear expression of PAX-8 (PAX-8 immunostaining, 200x). (E) CD10 immunostaining of the tumor cells. The renal nature of the tumor cells is supported by focally strong membranous positivity for CD10 (CD10 immunostaining, 200x). (F) CAIX immunostaining of the tumor cells. A focally positive membranous expression of carbonic anhydrase IX by tumor cells further supports its renal nature (CAIX immunostaining, 200x).

The Orthopedic Surgery team was consulted for surgical evaluation, which requested a bone biopsy of the femoral lesion. However, given the extensive lysis of the cortex and high risk for fracture with percutaneous biopsy, the Interventional Radiology team declined to biopsy. A week later, the patient returned to the Emergency Department with dramatically worsening left hip pain and was found to have a pathological intertrochanteric left femur fracture (Figure 2). The Orthopedic Surgery team decided on intramedullary screw fixation after consulting the Interventional Radiology team for a preoperative bone lesion embolization, with the intent to reduce intraoperative bleeding. A successful preoperative embolization was completed with near-complete occlusion of feeding neoplastic vessels (Figure 2). The patient was taken to the Orthopedic Surgery Department the following day for a successful intramedullary rod and screw fixation procedure in which no blood transfusion was required. During the operation, bone fragments were obtained and sent to pathology for evaluation, confirming the diagnosis of metastatic RCC (Figure 3). The patient was discharged from the hospital a week later and began palliative chemotherapy treatment shortly after her discharge.

## Discussion

This case reported a patient with RCC who underwent a successful preoperative embolization procedure, which allowed for decreased intraoperative hemorrhaging the following day when the patient underwent an intramedullary rod and screw fixation procedure. This case report serves as a reminder and adds to the existing literature that preoperative embolization is effective in reducing intraoperative bleeding in surgeries involving bone metastases.

RCC is the seventh most common cancer in the developing world, accounting for 2.2% of all cancers, with rates nearly doubling in developed countries in the last 50 years [7,8]. Nonmodifiable risk factors for RCC include older age (>60 years), race (African Americans, Hispanic Americans, and Native Americans), and male gender, with approximately 66% of RCC diagnoses made in males [8]. Modifiable risk factors include smoking, obesity, diet and alcohol, hypertension, and occupational exposure to drugs [8]. Approximately one-third of patients diagnosed with RCC will have bone metastasis at the time of diagnosis, while two-thirds will have bone metastasis within 24 months after diagnosis [9]. Even with early surgical resection, 20-50% will progress to metastatic disease, with a five-year survival rate of only 12% [8]. In the case presented, the patient displayed some of these characteristics described, as the patient was African American, had a nonmodifiable risk factor for RCC, had a smoking history, had a modifiable risk factor for RCC, and developed bone metastasis, a common outcome of RCC.

Surgery for bone metastasis can result in a significant intraoperative hemorrhage and increased transfusion requirements during surgery. A recent meta-analysis by Chen et al. reported an average intraoperative blood loss of 2,180 mL, with catastrophic blood loss as high as 5,000 mL, for metastatic spinal disease [10]. Procedures involving intramedullary stabilization of femoral bone metastases have also led to high blood transfusion requirements due to significant blood loss [11]. Intraoperative blood loss can lead to increased operative times, severe intraoperative complications, and delayed wound healing. Blood loss during surgery increases the risk of intraoperative mortality in surgically treated femoral bone metastases [1]. Moreover, an increased occurrence of postoperative infections has been associated with blood transfusions, with rates as high as 15% following femoral bone metastasis [12].

Since preoperative embolization was first introduced in 1975, it has been shown to decrease intraoperative bleeding, allowing for decreased rates of required blood transfusion and better surgical outcomes [13]. Bone tumors that are often aided by preoperative embolization are commonly hypervascular, such as giant cell and renal cell tumors [13]. A successful preoperative embolization is the reduction of >70% of the vascularization, performed within 72 hours of surgery [6]. A case-control study conducted in 2016 reported a less amount of intraoperative blood loss and blood transfusion requirements in patients who received a preoperative embolization procedure for a primary bone tumor versus those that did not [13]. Geraets et al. performed a systematic review that showed supporting evidence for preoperative embolization of RCC metastasis but not those from other primary tumors, including thyroid metastasis [6]. In the case reported, preoperative embolization proved to be beneficial in reducing intraoperative hemorrhaging as described in the literature.

## Conclusions

In this case, the Orthopedic Surgery team decided to perform a palliative intramedullary and screw fixation procedure after the patient was diagnosed with a pathological intertrochanteric left femur fracture. However, the presence of a left femoral lesion, presumed to be recurrent metastatic RCC, in the patient resulted in concern of intraoperative hemorrhage. The Interventional Radiology team was consulted to perform a preoperative bone lesion embolization to decrease intraoperative bleeding. The bone lesion was successfully embolized, allowing the patient to have a successful surgery the following day with no need for blood transfusions. This case represents the importance of a multidisciplinary approach to the treatment of bone metastasis with the intention of reducing morbidity and mortality.
